# Special Issue on “The Application of Metabolomics in Clinical Practice: Challenges and Opportunities”

**DOI:** 10.3390/metabo12040296

**Published:** 2022-03-28

**Authors:** Michele Mussap

**Affiliations:** Laboratory Medicine, Department of Surgical Sciences, School of Medicine, University of Cagliari, 09042 Monserrato, Italy; mumike153@gmail.com

This Special Issue aimed to collect studies based on clinical applications of metabolomics in human disease. Patient care is currently switching from the reductionist approach to one of precision medicine that is characterized by the collection of individual longitudinal clinical data and the use of multi-omics profiling and tailored therapeutic protocols. Diseases are no longer seen as the sum of structural and functional multiorgan damage and complications; rather, they are evaluated with regard to their full spectrum of associated phenotypic abnormalities caused by multiple factors, such as genetic and epigenetic changes, the pathogenesis of the disease, the host immune response, the gut microbiota, the microenvironment, and both the beneficial and adverse effects of any therapeutic interventions used. In this context, metabolomics plays a strategic role in depicting the individual molecular phenotype and discovering insights into cellular metabolic processes. Recently, metabolomics has moved far beyond the status of an emerging field within the “omics” sciences; actually, hundreds of research studies have utilized metabolomics to evaluate the metabolic profile of biological fluids and tissues, decipher alterations in metabolic pathways due to disease or specific physiological conditions (e.g., competitive sport, pregnancy), and measure the concentration of metabolites. A recent, elegant expression sounds as “metabolomics is the stethoscope of the 21st century” [1]. However, translating metabolomics from research to the routine clinical setting is a challenging perspective and the road ahead still seems to be long [2]. One of the most significant barriers slowing the transition is the heterogeneity of results between equivalent research studies. Consequently, results are often ambiguous or inconclusive. Clinicians and general practitioners need reliable results for routine patient care, and even metabolomic results must induce clinical decision-making, similar to any other laboratory test result. For example, quantitative data acquisition needs to be performed; this is a prerequisite for clinical application and decision-making [3]. All the variables associated with data should be standardized, including data description (univocal nomenclature), safety, storage conditions, data access regulatory rules, and the uniformity of measurement units [4,5]. The introduction of metabolomics into routine clinical practice is challenging for clinical laboratory services. Similar to any other laboratory test, metabolomics follows a complex process constituting several steps, the so-called brain-to-brain loop, which must meet rigorous qualitative criteria [6]. Each step can be managed by skilled laboratory professionals. From the pre-analytical phase, including the test request appropriateness, to the post-analytical phase, including test result interpretation, the quality of the total testing process is crucial for delivering reliable test results and minimizing errors [7]. A functional tool may be the introduction of laboratory-developed tests (LDT) based on metabolomics, as suggested by Lichtenberg et al. [8]. The LDT is a subset of in vitro diagnostic devices (IVDs) designed, manufactured, and used within a single laboratory. LDT may accelerate the transition of research metabolomics to routine clinical metabolomics; however, moving metabolomics from experimental research to routine clinical practice cannot imply the mere utilization of a given device or analytical platform. Rather, slotting metabolomics into clinical practice needs much more than the utilization of any easily available and simple laboratory method, for example including the conversion of metabolic patient-specific data into actionable clinical applications.

The aim of this Special Issue of *Metabolites* is to promote the transition of metabolomics from research to the clinical setting with the contribution of original data and data obtained from a literature review. Beyond concerns on harmonization between metabolomics-based studies, the review of Aderemi et al. [9] reported results emerging from the current literature on metabolomics-based diagnosis, treatment, and prognosis prediction in four non-communicable diseases—namely, Parkinson’s disease, diabetic retinopathy, Alzheimer’s disease, and cardiovascular (CV) disease—and in the screening of inborn errors of metabolism in children. In Parkinson’s disease, metabolomics have identified significant perturbations in the tryptophan metabolism and kynurenine pathway, with the accumulation of quinolinic acid; moreover, seven long-chain acylcarnitines were identified as candidate biomarkers. Various plasma metabolites were proposed as candidate biomarkers for diabetic retinopathy; interestingly, the analysis of vitreous humor unveiled significant perturbations in glucose and purine metabolism, confirming the presence of oxidative stress associated with diabetic retinopathy. In patients with CV disease, metabolomics allowed the identification of metabolites closely associated with the mortality rate among patients with acute heart failure (citrate, tyrosine, and 2- and 3-hydroxybutyrates); moreover, it was found that acetylglycine, threoninyl-glycine, glutarylglycine, and nonanoylcarnitine significantly discriminate between acute myocardial infarction (AMI) with fragmented QRS and AMI without QRS. Another study found alterations in the fatty acid metabolism of patients with chronic heart failure compared with healthy controls [10]. In particular, acylcarnitines and biogenic amines were significantly increased, and glycerophospholipids, sphingomyelins, and amino acids significantly decreased. The implantation of left ventricular assistance devices induced the normalization of the amino acid level in ischemic cardiomyopathy but not in dilative cardiomyopathy. In a large cohort of obese individuals aged 5–85 years, a metabolomics-based study found that glycine, leucine, arginine, valine, and acylcarnitines discriminate subjects with a high visceral adiposity index (VAI) and low bone mineral density (BMD) from those with normal VAI and BMD [11]. These gender-dependent results confirm that amino acids and carnitines play a crucial role in bone health and remodeling. An in vitro study conducted on Raji B Lymphoma Cell lines demonstrated the influence of the Zn ion treatment on cellular metabolism, resulting in a significant increase in glycerophosphocholine and fatty acids [12]. Notably, only arachidonic acid promoted cell apoptosis via the activation of caspase-3, and the addition of this polyunsaturated fatty acid led to mitochondrial permeability transition, which in turn releases cytochrome c. Metabolomics could elucidate the molecular mechanisms associated with the pathogenesis, severity, and effectiveness of the therapy for autoimmune diseases. For example, the severity of the disease can be mitigated by blocking glycolysis and glutaminolysis. The identification of changes in the metabolic profile of patients with rheumatoid arthritis treated with methotrexate allows clinicians to evaluate the therapeutic effectiveness and identify responders and non-responders [13]. Before initiating therapy with methotrexate, the plasma metabolome of patients with rheumatoid arthritis is marked by an increase in fatty acids, sphingomyelins, and phosphatidylcholines, in conjunction with a decrease in triglycerides and amino acids. The normalization of the plasma metabolome mirrors the effectiveness of methotrexate therapy after 16 weeks of therapy. In addition, metabolites involved in the nucleotide and energy metabolism, such as hypoxanthine, inosine, itaconic acid, and nicotinamide, differentiate responders and non-responders [13]. In an animal model of rheumatoid arthritis treated with methotrexate, the effectiveness of the therapy was marked by an increase in plasma quinolone in conjunction with a decrease in N-methylisoleucine [14]. Further pieces of evidence on the role of metabolomics for the identification of candidate biomarkers in severe autoimmune diseases, such as multiple sclerosis, rheumatoid arthritis, and systemic lupus erythematosus, were reported by Yoon et al. [15]. The skin surface lipidome was investigated in patients with Parkinson’s disease and patients with Alzheimer’s disease [16]. In Parkinson’s disease, the lipidome reflects the hyperactivation of sebum secretion, with an increase in most lipid components; conversely, in Alzheimer’s disease, the decrease in vitamin E and fatty alcohol 14:0 discriminates patients from healthy subjects. An original, exciting paper on neonatal nutrition showed a close relationship between the type of feeding (breastmilk and a mixture of breastmilk and commercial formulas) and the urine metabolome of infants [17]. In exclusively breastfed infants, the urine metabolome reflects the metabolic composition of the mother’s milk—in particular, the composition of oligosaccharides; this match is not present in infants fed with mixed methods. This finding has been related to the influence of human milk oligosaccharides on the gut microbiota development in very early infancy. Several studies on autism spectrum disorder (ASD) have used the metabolomic approach to investigate the metabolic fingerprint associated with the disease [18]. Recently, a metabolomics-based study demonstrated that the urine metabolome of autistic children reflects the severity of the disease, as assessed by standard clinical tools [19]. In this Special Issue, Piras et al. investigated the urine metabolic profile of a group of autistic children and their siblings [20]. The most relevant finding was the strong influence of increased intestinal permeability (leaky gut) on the urine metabolome. In detail, fucose, phenylacetylglycine, nicotinurate, and 1-methyl-nicotinamide strongly discriminated the urine metabolome of autistic children with leaky gut from that of the remaining autistic children. In a large cohort of critically ill patients, a metabolomics-based study investigated the sex-specific changes in the plasma metabolome following the oral administration of high doses of 25(OH)D (25-Hydroxy-vitamin D) [21]. Interestingly, three days after the start of the 25(OH)D intake, only females showed a significant correlation between 25(OH)D and the plasma level of long-chain acylcarnitines. In males but not in females, the increased 25(OH)D concentration induced a significant decrease in the plasma abundance of ceramides, dicarboxylate fatty acids, long-chain fatty acids, and polyunsaturated fatty acids. Subjects showing an increase in 25(OH)D > 7.5 mg/L were considered responders; in this cohort, the number of branched-chain amino acids and short-chain acyl-carnitines was significantly lower in women than in men. This finding demonstrates that specific pharmacometabolic differences between men and women are reflected by specific metabolic signatures. In conclusion, we hope that this Special Issue could significantly accelerate the transition of metabolomics from research to routine clinical practice.
